# Non-Photochemical Quenching Involved in the Regulation of Photosynthesis of Rice Leaves under High Nitrogen Conditions

**DOI:** 10.3390/ijms21062115

**Published:** 2020-03-19

**Authors:** Amara Cisse, Xia Zhao, Weimeng Fu, Romesh Eric Romy Kim, Tingting Chen, Longxing Tao, Baohua Feng

**Affiliations:** 1 National Key Laboratory of Rice Biology, China National Rice Research Institute, Hangzhou 310006, China; cisseam@ymail.com (A.C.); zhaoxia288288@163.com (X.Z.); fuwmeng@163.com (W.F.); thekingmbembe@gmail.com (R.E.R.K.); ntchtt@163.com (T.C.); 2 Yibin University, Yibin 644000, China

**Keywords:** rice, photosynthesis, nitrogen, non-photochemical quenching

## Abstract

Excess and deficient nitrogen (N) inhibit photosynthesis in the leaves of rice plants, but the underlying mechanism is still unclear. N can improve the chlorophyll content and thus affect photon absorption, but the photosynthetic rate does not increase accordingly. To investigate this mechanism, three concentrations of N treatments were applied to two rice varieties, Zhefu802 and *Fgl*. The results indicated increased chlorophyll content of leaves with an increased N supply. Little discrepancy was detected in Rubisco enzyme activity and Non-photochemical quenching (NPQ) in the high nitrogen (HN) and moderate nitrogen (MN) treatments. The model that photoinhibition occurs in Zhefu802 due to a lack of balance of light absorption and utilization is supported by the higher malondialdehyde (MDA) content, higher H_2_O_2_ content, and photoinhibitory quenching (qI) in HN treatment compared with MN treatment. A lower proportion of N in leaf was used to synthesize chlorophyll for *Fgl* compared with Zhefu802, reducing the likelihood of photoinhibition under HN treatment. In conclusion, HN supply does not allow ideal photosynthetic rate and increases the likelihood of photoinhibition because it does not sustain the balance of light absorption and utilization. Apart from Rubisco enzyme activity, NPQ mainly contributes to the unbalance. These results of this study will provide reference for the effective N management of rice.

## 1. Introduction

Nitrogen (N) is the most important nutrient element in plants [1,2], and is present in proteins, nucleic acids, co-enzymes, and membranes, with important roles in metabolism. N metabolism is closely related to electronic transport in chloroplasts [3]. The allocation of N in the photosynthetic apparatus affects the photosynthetic rate and the photosynthetic N use efficiency [4,5,6]. However, a discrepancy between N content and photosynthetic rate has been observed, which may reflect altered photosynthetic efficiency caused by changes in CO_2_ concentration [7].

Photosynthesis is a basic physiological process in plants, which transforms solar energy to chemical energy. Light is required for photosynthesis, but excessive light energy absorbed by pigment molecules can disrupt the oxidative balance in plants and inhibit photosynthesis, especially in C_3_ plants [8]. In C_3_ plants, leaf photosynthesis only utilizes ∼25% of sunlight energy absorbed by pigment molecules for the photosynthetic metabolism of NADPH and ATP [9,10]. Excess irradiated energy will inactivate the reaction center, resulting in 3P680^*^, a triple state of the chlorophyll molecules [11]. The triple molecules can react with O_2_ to generate deleterious singlet O_2_ (^1^O_2_) [12], degrade the PSII reaction center protein D1, inactivate PSII and inhibit photosynthesis [13]. To eliminate damage from excessive light, plants have evolved some photoprotective mechanisms [7]. One mechanism is to dissipate the excessive excitation energy as heat in PSII antenna complex, which is known as non-photochemical quenching (NPQ) of chlorophyll fluorescence [14,15]. Although ubiquitous, the role of NPQ in plant productivity remains uncertain because it momentarily reduces the quantum efficiency of photosynthesis. Hubbart et al. [16] assessed photoprotection in rice at the whole canopy scale, and demonstrated that compared to wild-type plants, the overexpression of *PsbS* resulted in higher NPQ, increased canopy radiation use efficiency, and grain yield in fluctuating light. Genotypic variation of NPQ in rice was observed under natural solar radiation [17]. Wang et al. [8] reported that *OsPsbS1* explains more than 40% of the NPQ variation. According to the induction speed of the relaxation kinetics curve in the dark, NPQ can be divided into three main components: qE, energy-dependent quenching, or fast NPQ, which is induced in seconds and is based on the proton gradient across the thylakoid membrane; qT, state transition quenching, or middle NPQ, which is induced in minutes; and the third component, qI, photo-inhibition quenching, or slow NPQ, which is induced very slowly [18,19]. The major photoprotective strategy is qE, where the driving step is the formation of ΔpH across the thylakoid membrane [20]. The decreased pH in the thylakoid lumen often activates the PsbS protein and violaxanthin de-epoxidase, which converts violaxanthin to zeaxanthin [19,21]. These activated proteins then bind to the light harvesting complex (LHC) polypeptides, induce conformational changes in these proteins, and then trigger qE. When the thylakoid membrane is exposed to high light, the domains of LHCII and PSII reaction centers can cluster together [22,23]. Johnson et al. [24] found that these structural changes occurred within five minutes and were enhanced by the de-epoxidation of violaxanthin to zeaxanthin.

N metabolism is proposed to play a significant role in the photosynthetic acclimation of plants [25,26]. Rice with N-deficiency is more sensitive to photo-inhibition [27,28]. Under N-deficiency, decreasing light energy absorption and increasing induction of NPQ alleviate the reducing capacity in PSII [29,30]. Chen et al. [31] reported that PSII in the grape leaf was easily oxidized by high light and enhanced NPQ induction with N-deficiency. An increased ratio of carotenoid to chlorophyll can prevent the production of singlet O_2_ (^1^O_2_) [32,33]. The NPQ inducted by N deficiency is mainly qE NPQ, and not qI NPQ [34]. NPQ induction can improve rice adaption to N concentrations by the consumption of excess light energy. Shrestha et al. [35] reported the close relationship of NPQ induction to the N nutrient situation in rice, and considered PRI (photochemical reflectance index), which indicates the oxidation of xanthophylls, a reliable index to estimate the N nutrient status in a rice field. However, the role of N in NPQ induction still remains elusive. In this study, two rice genotypes, Zhefu802 and the near isogenic line *Fgl* with a faded green leaf phenotype, were subjected to three N concentration treatments. The results provide insight into the mechanisms underlying the influences of N on NPQ induction, light energy utilization, and photosynthesis, as well as a new insight in N use efficiency.

## 2. Results

### 2.1. Chlorophyll Content and Dry Matter Weight

**Figure 1:**
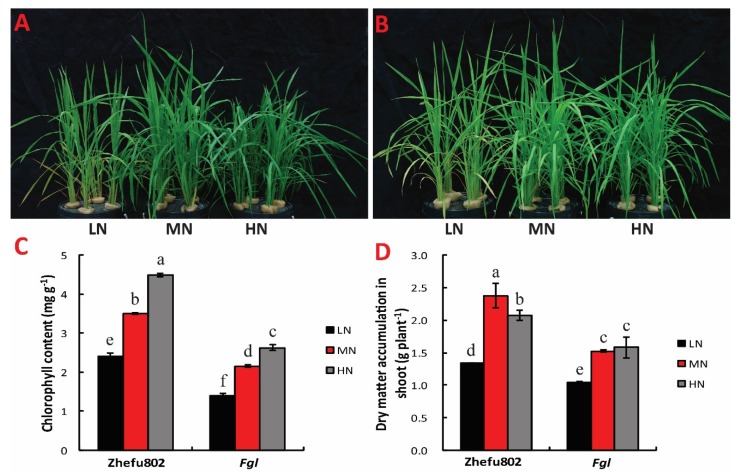
Effect of 20-day nitrogen treatments at sixth leaf stage on the growth of Zhefu802 (**A**) and *Fgl* (**B**), and chlorophyll content (**C**), dry matter accumulation (**D**). LN, low nitrogen, 10 mg L^−1^; MN, moderate nitrogen, 40 mg L^−1^; HN, high nitrogen, 160 mg L^−1^. Different letters denote significant difference as determined by LSD test (*p* < 0.05, *n* = 3).

N treatments significantly influenced chlorophyll content and dry matter accumulation. Leaf color was darker with increased N concentration in both cultivars (Figure 1A,B). Chlorophyll content increased with increased N concentration. For Zhefu802, increases of about 35% and 65% were observed in the MN and HN treatments, respectively, compared with the LN treatment, while only 25% and 34% was observed in *Fgl* (Figure 1C).

For Zhefu802, HN and LN significantly decreased the over-ground dry matter by 15% and 24% respectively compared with MN. For *Fgl*, LN significantly decreased the over-ground dry matter by 40%, but there was no significant difference between MN and HN treatments (Figure 1D).

### 2.2. The A-Ci Curve

**Figure 2:**
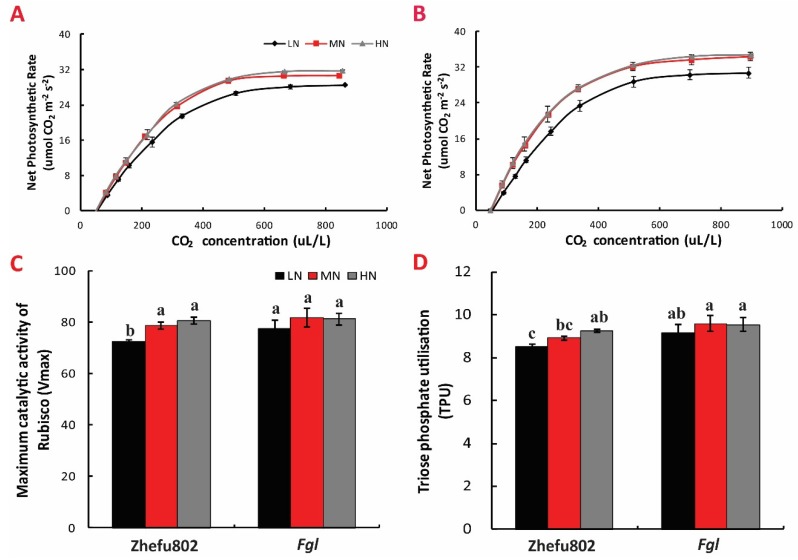
Effect of nitrogen treatments on photosynthetic CO_2_ response curve of Zhefu802 (**A**) and *Fgl* (**B**). (**C**) Maximum catalytic activity of Rubisco (V_max_), (**D**)Triose phosphate utilisation (TPU). LN, low nitrogen, 10 mg L^−1^; MN, moderate nitrogen, 40 mg L^−1^; HN, high nitrogen, 160 mg L^−1^. Different letters denote significant difference as determined by LSD test (*p* < 0.05, *n* = 3).

There was no significant difference in the *A-Ci* curves betwwen HN and MN treatment, but the curves of HN and MN treatments were markedly higher than those of LN for both cultivars. Under the same N treatment, the *A-Ci* curve in *Fgl* was significantly higher than that for Zhefu802 (Figure 2A,B). 

The *A-Ci* curve fitting parameters including V_max_ (maximum catalytic activity of Rubisco), and TPU (triose phosphate utilisation) showed no significant differences among the three N treatments in *Fgl*, but in Zhefu802, LN treatment significantly decreased the Vmax and TPU compared to MN and HN treatments (Figure 2C,D).

### 2.3. Rubisco Activity and N Content

**Figure 3:**
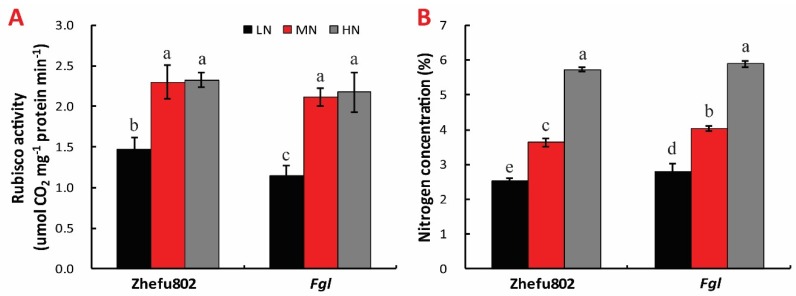
Effect of nitrogen treatments on Rubisco activity (**A**) and nitrogen concentration (**B**) in leaf. LN, low nitrogen, 10 mg L^−1^; MN, moderate nitrogen, 40 mg L^−1^; HN, high nitrogen, 160 mg L^−1^. Different letters denote significant difference as determined by LSD test (*p* < 0.05, *n* = 3).

Activity of Rubisco enzyme was substantially inhibited by LN compared with MN in both cultivars. However, no significant difference was observed between MN and HN for both cultivars (Figure 3A). The N content in the leaf significantly increased with increased N treatment concentration in both cultivars. Increases of about 35% and 45% relative to LN were measured in Zhefu802 with MN and HN treatments, respectively, with 32% and 42% increases in *Fgl* (Figure 3B).

### 2.4. Chlorophyll Fluorescence

**Figure 4:**
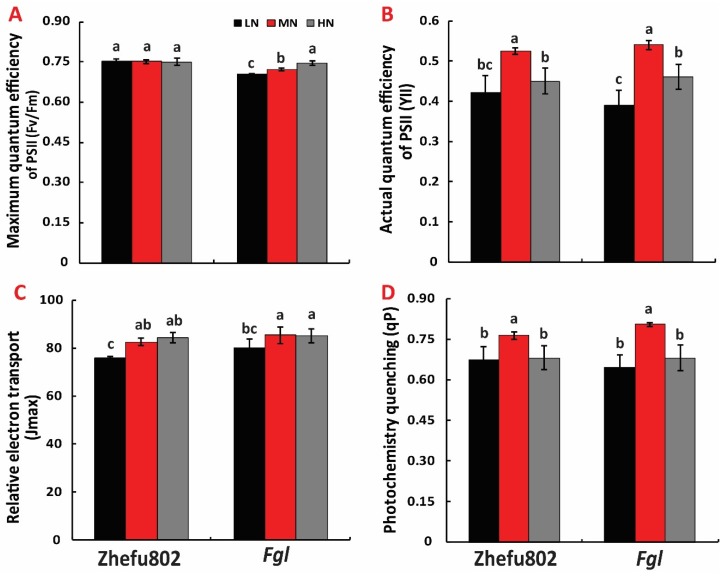
Photosynthesis and chlorophyll fluorescence parameter in response to different nitrogen treatments. (**A**) Maximum quantum efficiency of PSII (F_v_/F_m_), (**B**) Actual quantum efficiency of PSII (YII), (**C**) Maximum electron transport rate (J_max_), (**D**) Photochemistry quenching (qP). LN, low nitrogen, 10 mg L^−1^; MN, moderate nitrogen, 40 mg L^−1^; HN, high nitrogen, 160 mg L^−1^. Different letters denote significant difference as determined by LSD test (*p* < 0.05, *n* = 3).

No significant difference was observed in F_v_/F_m_ among the three N treatments in Zhefu802, but significant differences were observed in *Fgl*. The HN resulted in the highest F_v_/F_m_, followed by MN, and LN was the lowest (Figure 4A). The MN resulted in a significantly higher YII than LN and HN in both cultivars. No significant difference was observed in YII between LN and HN in Zhefu802, while in *Fgl*, LN significantly decreased the YII when compared to the HN treatment (Figure 4B). Measurement of the maximum electron transport rate (J_max_) revealed that LN significantly decreased the Jmax compared to the MN and HN treatments for both cultivars (Figure 4C). However, MN showed a significantly higher qP than LN and HN in both cultivars (Figure 4D).

### 2.5. NPQ Induction and Relaxation Analysis

**Figure 5:**
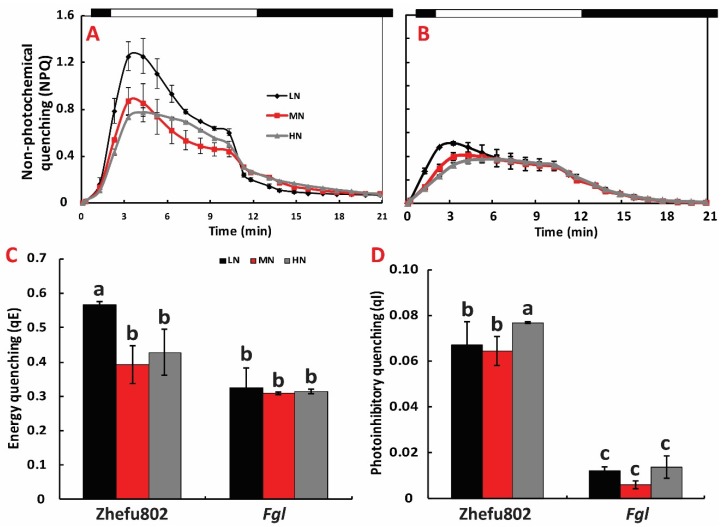
NPQ induction and relaxation analysis of two rice cultivars under different nitrogen treatments. (**A**) NPQ induction of Zhefu802, (**B**) NPQ induction of *Fgl*, (**C**) energy quenching(qE), (**D**) photoinhibitory quenching (qI). LN, low nitrogen, 10 mg L^−1^; MN, moderate nitrogen, 40 mg L^−1^; HN, high nitrogen, 160 mg L^−1^. Different letters denote significant difference as determined by LSD test (*p* < 0.05, *n* = 3).

Compared to the MN treatment, LN significantly increased NPQ induction and HN decreased NPQ induction for both cultivars. However, HN significantly slowed the decrease after the NPQ peak value in Zhefu802 (Figure 5A,B). The relaxation analysis revealed that LN significantly increased qE by 44.04% compared to the MN treatment in Zhefu802, while no significant difference was observed in qE between HN and MN for Zhefu802. In *Fgl*, no significant difference was observed in qE between N treatments (Figure 5C). No significant difference in qI values were observed for N treatments in *Fgl*, but in Zhefu802, qI was significantly higher for HN compared to that under MN (Figure 5D).

### 2.6. H_2_O_2_ and MDA Content

**Figure 6:**
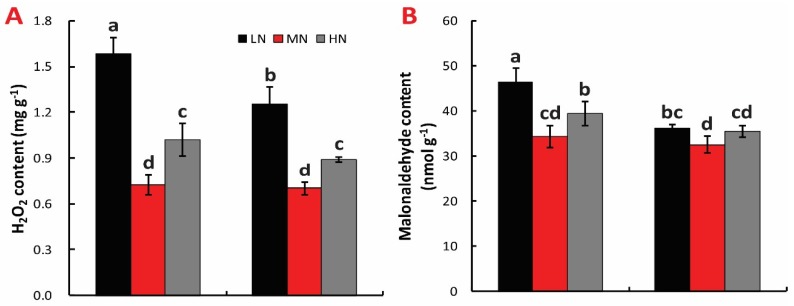
Effect of N treatment on H_2_O_2_ content (**A**) and malonaldehyde content (**B**) in leaf. LN, low nitrogen, 10 mg L^−1^; MN, moderate nitrogen, 40 mg L^−1^; HN, high nitrogen, 160 mg L^−1^. Different letters denote significant difference as determined by LSD test (*p* < 0.05, *n* = 3).

LN and HN significantly increased the H_2_O_2_ content in both cultivars, with increases of 118.5% and 41.02%, respectively, relative to MN in Zhefu802. In *Fgl*, only 79.06% and 26.99% increases were observed in LN and HN, respectively, compared to MN (Figure 6A). The change of MDA content showed the same tendency in both cultivars. MDA content was higher under LN and HN compared with MN treatment. LN significantly increased the MDA content by 28.47% and 13.62%, respectively, in Zhefu802 and *Fgl* varieties. HN significantly raised the MDA content in Zhefu802 compared with MN treatment, while no significant difference was observed in *Fgl* for these conditions (Figure 6B).

### 2.7. Antioxidant Activity

**Figure 7:**
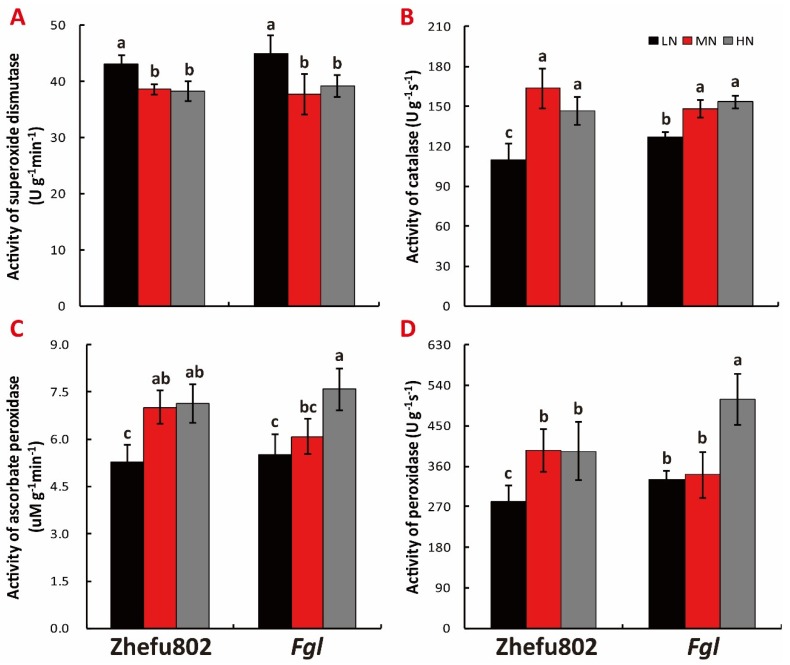
Effect of nitrogen treatments on superoxide dismutase (**A**, SOD), catalase (**B**, CAT), ascorbate peroxidase (**C**, APX) and peroxidase (**D**, POD). LN, low nitrogen, 10 mg L^−1^; MN, moderate nitrogen, 40 mg L^−1^; HN, high nitrogen, 160 mg L^−1^. Different letters denote significant difference as determined by LSD test (*p* < 0.05, *n* = 3).

LN significantly increased the superoxide dismutase (SOD) activity in both cultivars compared with the MN treatment, with no significant difference between HM and MN treatment (Figure 7A). However, LN significantly deceased the catalase (CAT) activity in both cultivars compared to MN treatment, while no significant difference between HM and MN treatment was determined. Lower CAT activity in Zhefu802 was observed compared to that in Fgl under LN treatment (Figure 7B). Figure 7C showed significant decrease of peroxidase (POD) activity in Zhefu802 under LN treatment compared with the MN and HN treatments. In *Fgl*, HN significantly promoted POD activity compared with the LN and MN treatments. The ascorbate peroxidase (APX) activity showed the same trend as POD activity in Zhefu802, with significant suppression by LN of APX activity, and in *Fgl*, HN significantly increased APX activity (Figure 7D).

### 2.8. Pigments Involved in the Xanthophyll Cycle

As shown in Table 1, xanthophyll cycle components including V, A, Z, Z + 1/2A decreased with increased N concentration in both cultivars except for the V content in *Fgl* under HN treatment. LN increased the Z + 1/2A content by 17.27% and 8.81% in Zhefu802 and *Fgl,* respectively, compared with the MN treatment. However, the HN treatment decreased the Z + 1/2A content by 6.37% and 4.63% in Zhefu802 and *Fgl,* respectively, compared with the MN treatment. LN significantly increased the β-Carotene content in *Fgl* compared to the MN and HN treatments, but in Zhefu802, no significant difference was observed between the LN and MN or between the HN and MN treatments. β-Carotene content in *Fgl* was significantly higher than that in Zhefu802 under all treatments (Table 1).

### 2.9. Structure of Mesophyll Cell

**Figure 8:**
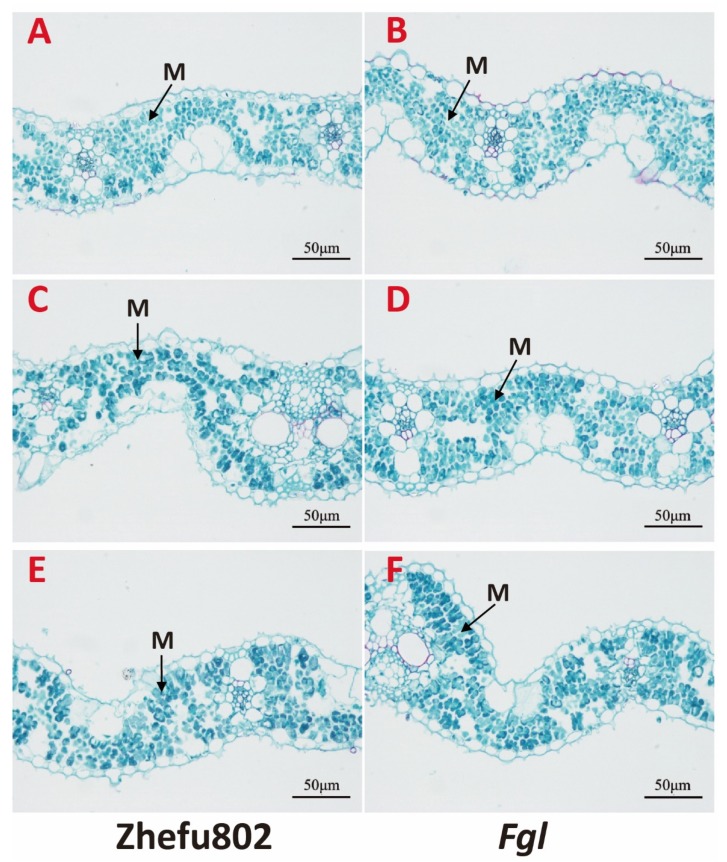
Effect of nitrogen treatments on cross-sections of the youngest fully expanded leaves. (**A**,**C**,**E**) Cross-section samples of Zhefu802; (**B**,**D**,**F**) Cross-section samples of *Fgl*; (**A**,**B**) LN treatments; (**C**,**D**) MN treatments; (**E**,**F**) HN treatments. M: mesophyll tissue.

Different N concentration treatments significantly influenced leaf structure. The number and size of the mesophyll cells significantly impacted the light intensity and the CO_2_ transport in the interior space of leaf. As Figure 8 shows, LN treatment significantly reduced the volume of the leaf mesophyll but increased the number of mesophyll cells in both cultivars compared with the MN treatment (Figure 8A–D). However, there was no significant difference in either the size and number of the leaf mesophyll between the HN and MN treatments (Figure 8C–F). Furthermore, no significent difference was found between the two cultivars under the same treatment.

## 3. Discussion

### 3.1. High N Supply does not Lead To Ideal Photosynthetic Rate and Raises Likelihood of Photoinhibition

Yield and biomass do not increase with N application, and N use efficiency decreases with increased N application, largely due to photosynthesis [4,5]. Most (75–80%) nitrogen in leaf exists as enzymes involved in photosynthesis [36]. N content per leaf area explains 35% of the genetic variation of photosynthesis in rice [37], the allocation of N in the photosynthetic mechanism directly impacts photosynthesis and N use efficiency [6]. In sugarcane plant, N increased the photosynthetic properties by increasing the chlorophyll content, Rubisco enzymes activation, sugar content and photosynthesis-related metabolites [38]. In this study, the leaf N concentration in *Fgl* was significantly higher than that in Zhefu802, except for under HN treatment. However, higher chlorophyll content was detected in Zhefu802 compared to the level in *Fgl* under all treatments, especially for HN treatment. Chlorophyll content in Zhefu802 was 70.71% higher than that in *Fgl* under HN treatment. This suggested that a lower proportion of N in leaf was used to synthesize chlorophyll for *Fgl* compared to Zhefu802 under HN treatment. Elevated chlorophyll, a main component of light harvesting compound and the light reaction center, increases light energy capture and absorption. Rubisco enzyme content, a main form of N reserve in leaf, increases with N application. However, both the results from previous works and those from this study showed that HN treatment did not enhance Rubisco enzyme activation. For Zhefu802, HN treatment likely substantially improves the light absorption capability but does not obviously change CO_2_ assimilation or light utilization. In this condition, the generation of excess irradiated energy can occur. Excess irradiated energy of photosystems can inactivate the reaction center, resulting in the production of reactive oxygen species [39,40]. NPQ is a photoprotective mechanism that can eliminate excess irradiated energy. It has been well documented that NPQ was increased immediately by stresses which played an important role in the reduction in electron transport, increasing in heat dissipation, and finally increasing in rice resistance to abiotic stresses including chilling, salt, and heat [32,41,42]. However, in our study, little change was observed for HN and MN treatments. The levels of pigments involved in the xanthophyll cycle were significant lower under HN treatment compared with MN treatment. Xanthophyll cycle plays an important role in regulating NPQ induction. Zhu et al. [43] reported that exogenous ABA resulted in a rise in the level of NPQ by altering the kinetics of de-epoxidation of the xanthophyll cycle, and increased resistance to NaCl stress in rice. Besides xanthophyll cycle, ΔpH across thylakoid membrane, PsbS protein, and other element could regulate NPQ, so there is a possibility that the levels of pigments involved in the xanthophyll cycle is inconsistent with induction of NPQ. Summing up the above, the high chlorophyll content in Zhefu802 due to HN treatment leads to the presence of excess irradiated energy, which cannot be used by CO_2_ assimilation or cleared by NPQ. This hypothesis is supported by the observed higher MDA content, H_2_O_2_ content, and qI of leaf in plants subjected to HN treatment compared to those subjected to MN treatment. Photoinhibition is the result of an imbalance of light absorption and utilization, preventing high photosynthesis rates under HN treatment. This imbalance can be affected by many factors, such as chlorophyll content, activation of the Rubisco enzyme, and NPQ. For *Fgl*, even though the activation of the Rubisco enzyme and NPQ induction were not elevated by HN treatment, the generation of excess irradiated energy was limited by the lower chlorophyll content under HN treatment. There was no obvious difference in the content of MDA or H_2_O_2_ between the HN and MN treatments. The lower hyperoxide content contributes to higher photosynthesis of *Fgl* under HN compared with Zhefu802. There is a significant difference in response mechanisms to different N levels between the two varieties. The chlorophyll levels of common rice cultivars are similar to that of Zhefu802. Rice subjected to a high N application cannot obtain the ideal photosynthetic rate, but is vulnerable to photoinhibition without the sustained balance of light absorption and utilization. Li et al. [44] reported that the activation of the Rubisco enzyme was a primary reason that higher photosynthesis was not obtained under HN treatment, but in this study, the results revealed that besides the rubisco enzyme, the little change of NPQ between HN and MN treatments is another main reason for this phenomenon. Brestič et al. [45] reported that wheat mutant “ANK” with low PSI content had a limited capacity to build up the transthylakoid proton gradient needed to trigger NPQ. At the same time, Repetitive light pulse-induced photoinhibition of PSI severely affects CO_2_ assimilation [46]. Therefore, performance of PSI is a potential possibility of the little change of NPQ and activity of rubisco enzyme, but the hypothesis needs to be demonstrated by further research.

### 3.2. Mechanism of Acclimation to N Insufficiency

N is the primary element of chlorophyll, Rubisco enzyme, and the electron transport chain. In this study, decreased net photosynthetic rate and biomass were observed under LN treatment. At the same time, there was less chlorophyll content and lower activation of Rubisco enzyme under LN treatment compared to MN treatment, consistent with the results achieved by Sun [47]. Increased H_2_O_2_ and MDA content and enhanced photoinhibition were observed under LN treatment. These results revealed that photosynthesis and the growth of rice were inhibited by a low N level. Chen et al. [28] obtained similar results in field studies. The induction of NPQ was markedly strengthened by LN treatment, especially for Zhefu802. The discrepancy in qE of Zhefu802 between LN and MN treatments was as high as 80.83%. There is genetic variation in the NPQ capacity [8], and a difference in NPQ capacity was found between Zhefu802 and *Fgl*. Significantly higher zeaxanthin content contributes to the induction of NPQ under LN treatment. Chen et al. [31] investigated the response of grapes on N treatments, and showed that compared to the sufficient N supply in field, grape with deficiency N exhibited more sensitive to the photoinhibition and enhanced induction of NPQ. Previous studies verified that decreased light absorption and the induction of NPQ are able to alleviate the excessive excitation energy in the photosystem. Brestič et al. [48] found that the thermal deactivation of energy in the photosystem II antennae appears to be the main protective mechanism against high-light damage in French bean leaves during drought conditions, whereas photorespiration does not protect the photosynthetic apparatus. Thus, strengthened NPQ is an important adaptive mechanism to N insufficiency in rice. Faseela et al. [49] demonstrated chlorophyll fluorescence parameters could be as indicators of a particular abiotic stress in rice. The low N obviously altered the leaf structure in both cultivars. Size reduction and an increased number of mesophyll cells were observed in both cultivars under LN treatment compared with the other two treatments. Smaller cell volume and more mesophyll cells would reduce light absorption by light scattering and strengthen CO_2_ transport [50]. Therefore, we hypothesized that these changes in photoprotection and leaf structure helped adaptation to N insufficiency.

## 4. Materials and Methods

### 4.1. Experimental-Set Up

The experiments were conducted at the China National Rice Research Institute (CNRRI) in Fuyang City, China. Two rice genotypes, Zhefu802, with a normal leaf color, and its near-isogenic line *Fgl*, with a pale green leaf color, were used in this experiment. The seeds were incubated at 30 °C for 48 h in the dark and geminated in a wetted towel at 35 °C for an additional 24 h. The germinated seeds were selected and transferred to a nursery containing water for a week, and then a half-strength Yoshida’s rice nutrient solution was substituted [51]. Next, the half-strength solution was used for one week before using the full-strength solution. The pH value was adjusted every three days and fresh solution was exchanged every five days. When the plants reached the 4-leaf stage, six plants were together transferred to a 5 L bucket filled with nutrient solution. Three N treatments were used: LN, 10 mg L^−1^; MN, 40 mg L^−1^ (the same as that in the original nutrient solution); and HN, 160 mg L^−1^. These treatments began when the plants reached the 6-leaf stage and treatment was maintained for 20 days. The plants were kept in the greenhouse under natural conditions with three replicates per treatment; each replicate included three buckets, and the entire experiment was performed in duplicate. The second expanded leaves of some plants in the same replicate were sampled immediately after the treatments, put into liquid N for two hours, and then stored at −80 °C for further analysis. The rest plants were conducted for A-Ci curve, chlorophyll fluorescence measurement and gas exchanges, and biomass weight.

### 4.2. Biomass Weight

After 20 days of treatment, 6 plants were randomly sampled. The plants were divided into roots, stem-sheaths, and leaves. A leaf area scanner Li-3100 (Li-Cor; Lincoln, NE, USA) was used to measure the leaf area. The samples were heated at 105 °C for 30 min, and then dried at 80 °C until the weight was steady. The specific leaf area was calculated as the ratio of the leaf area to the mass of the dry matter of the leaf.

### 4.3. Rubisco Activity Measurement

The rice leaf Rubisco activity was determined following the method described by Li et al. [44]. The youngest fully expanded leaves were sampled after the 20-day N treatment and stored in liquid nitrogen. About 0.2 g of leaf samples were homogenized with 4 mL extraction buffer containing 50 mM Tris-HCl (pH 7.5), 1 mM EDTA-Na_2_, 10 mM MgCl_2_, 12.5% (*v*/*v*) glycerine, 1% PVP-40, and 10 mM β-mercaptoethanol. The homogenate was centrifuged at 10,000× *g* for 15 min at 4 °C. The 0.1 mL supernate was added to a 0.9 mL reaction solution containing 55.56 mM HEPES–NaOH (pH 7.5), 11.11 mM NaHCO_3_, 20.22 mM MgCl_2_, 2.78 mM dithiothreitol, 1.11 mM EDTA-Na_2_, 11.11 U creatine phosphokinase, 11.11 U 3-phosphoglyceric phosphokinase, 1.11 U glyceraldehyde 3-phosphate dehydrogenase, 5.56 mM ATP, 0.17 mM NADH, 5.56 mM phosphocreatine, 0.67 mM ribulose diphosphate, and 11.11 mM Tris-HCl (pH 7.5). The absorbance at 340 nm of the solution was monitored for 90 s, and the slope was used to estimate the Rubisco activity.

### 4.4. N Content Measurement

The semi-micro Kjeldahl distilled method was used to measure the total N content in the leaf samples [52]. A sample of 0.2 g was ground into powder and was then digested by H_2_SO_4_-catalyzer.

### 4.5. A-Ci Curve Determination

We measured the CO_2_ response and plotted the assimilation rate against the intercellular CO_2_ concentration (*A-Ci* response curve). To do this, the plants were subjected at least for 20 min to a CO_2_ concentration of 400 μmol mol^−1^, a temperature of 30 °C (about 2 degrees above the environment), a PPFD of 1500 μmol m^−2^ s^−1^, and a relative humidity of 70−80%. Using photosynthesis measurement equipment (Li-6400; Li-Cor; Lincoln, NE, USA), measurement was made after N level treatment. Gas exchange parameters were recorded at a series of Ci values: 400, 300, 200, 150, 100, 50, 400, 600, 800, and 1000 µmol mol^−1^. When the leaves reached photosynthetic equilibration, the photosynthetic rate data was recorded every two seconds for two minutes. The maximum catalytic activity of Rubisco (Vmax) and maximum triose phosphate utilization (TPU) were calculated according to Sharkey et al. [53]. The *A-Ci* curve was drawn with the net photosynthetic rate as the ordinate, and the CO_2_ concentration as the abscissa.

### 4.6. Chlorophyll Fluorescence Measurement and Gas Exchanges

Chlorophyll fluorescence parameters were measured using a PAM2500 chlorophyll fluorometer (Walz Heinz GmbH, Effeltrich, Germany). After N level treatment, the plants were adapted in the darkness for 30 min. The minimum fluorescence (Fo) at open PSII centers was determined by measuring light, and the maximum fluorescence (Fm) at closed PSII centers was examined after the application of a 0.8 s pulse of saturating light (600 mol m^−2^s^−1^ white light) after 30 min of darkness. The maximum quantum efficiency of PSII (Fv/Fm) was defined as (Fm-Fo)/Fm. Actinic light was applied to measure the steady-state chlorophyll fluorescence (Fs). In the light-adapted state, Fm’ was measured by applying a saturating pulse, and Fo’ was measured by switching off the actinic light for 2 s after the saturating pulse and then applying far-red light. NPQ induction was calculated from the fluorometer readings. The actual quantum efficiency of PSII (YII) was defined as (Fm’-Fs)/Fm’, photochemistry quench (qP) was defined as 1-(Fs-Fo’)/(Fm’-Fo’), light-dependent regulative dissipation (Y(NPQ)) was defined as F/Fm’-F/Fm, and basal constitutive decay of excited chlorophyll (Y(NO)) was defined as F/Fm [54,55]. Typical quenching analysis followed by relaxation analysis was applied to the dark-adapted, youngest, fully-developed leaves using actinic light of 1200 μmol m^−2^ s^−1^. F′′m was defined as the maximal fluorescence during dark recovery after previous illumination. F′m and F′′m were determined after 10 min of illumination and after 10 min darkness recovery, respectively, and qE = Fm/F′m − Fm/F′′m; qI = Fm/F′′m-1 [56].

### 4.7. Chlorophyll and Xanthophylls Measurement

The youngest fully expanded leaves were sampled after the N treatment. The main leaf vein was removed, and the material was stored in liquid nitrogen before measurement. About 0.1 g leaf samples were homogenized in pure acetone. The xanthophyll cycle components including anhydrorhodovibrin, violaxanthin, zeaxanthine, and β-carotenoid were separated and measured by HPLC (HP1100, Agilent, PaloAlto, CA, USA) using a LiChrospher C18 column (Hypersil ODS 4.6 × 250 mm, 4.5 μm) as described by Havaux et al. [57]. Chlorophyll was extracted as described in Sartory and Grobbelaar [58]. Briefly, 3 cm^2^ samples of fresh leaf were immersed in 20 mL 95% alcohol for two days. Chlorophyll a and b concentration was measured using a spectrophotometer, and the following calculations were used: chlorophyll a (Ca μg mL^−1^) = 13.95(A665) − 6.88(A649), and chlorophyll b (Cb μg mL^−1^) = 24.96(A649) − 7.32(A665).

### 4.8. Mesophyll Cell Structure Observation

Leaves were sampled after the N treatment by fixing segments of leaf at 4 °C in 2.5% glutaraldehyde, removing the air with a vacuum pump, and then treated with 1% osmium tetroxide overnight at 4 °C. The segments were dehydrated in a graded acetone series and embedded in paraffin. After staining with safranine, the leaf cells were observed with a microscope (Leica DM1000, Leica Microsystems, Ltd., Germany), and electron micrographs were taken with a digital camera.

### 4.9. H_2_O_2_ and MDA Content Measurement

The H_2_O_2_ content was measured following the method of Brennan and Frenkel [59]. After the N treatment, the youngest fully expanded leaves (0.2 g) were ground in liquid nitrogen into powder and homogenized with 4 mL of 10 mM 3-amino-1,2,4-triazole. After centrifugation for 25 min at 6000× *g*, 1 mL of 0.1% titanium tetrachloride in 20% H_2_SO_4_ was added to 2 mL supernatant. The reaction solution was further centrifuged to remove the undissolved materials, and absorbance was recorded at 410 nm. The H_2_O_2_ concentration was calculated using a standard curve.

The MDA content was measured following the method of Dionisio-Sese and Tobita [60]. After N-treatment, the youngest fully expanded leaves (0.2 g) were ground in liquid nitrogen into powder and homogenized in 4 mL 10% (*w*/*v*) trichloroacetic acid. The homogenate was centrifuged for 15 min at 10,000 × g, and then 1.5 mL supernatant was added to the same volume of a 0.67% (*w*/*v*) thiobarbituric acid solution containing 10% (*w*/*v*) trichloroacetic acid. The mixture was heated at 100 °C for 30 min and then the reaction was halted by rapid transfer to an ice bath. The cooled reaction solution was then centrifuged at 10,000 × g for 10 min, and the absorbance of the supernatant was measured at 450, 532, and 600 nm. The MDA concentration was measured following the formulation: MDA (μM) = 6.45 × (OD_532_ − OD_600_) − 0.56 × OD_450_.

### 4.10. Antioxidant Enzyme Activity Measurement

After N- treatment, the youngest fully expanded leaves (0.2 g) were ground in liquid nitrogen into powder and homogenized with 4 mL of 100 mM phosphate buffer (pH 7.0). The homogenate was centrifuged for 15 min at 10,000× *g* at 4 °C, and then the supernatant was maintained at 4 °C for SOD, POD, APX, and CAT activity measurements. The SOD activity was measured as described by Giannopolitis and Ries [61]. The POD activity was determined as described by Maehly and Chance [62]. The CAT activity was measured according to the method of Aebi [63]. The APX activity was measured according to the method of Bonnecarrère et al. [64].

### 4.11. Statistical Analysis

Data were processed with SPSS I software (IBM Corp., Armonk, NY, USA) to determine the significance of the differences between or among the data. One-way analysis of variance (ANOVA) was conducted with a least significant difference test (LSD) at *p* ≤ 0.05. The mean values and standard errors in the figures represent data from three experimental replicates unless otherwise stated.

## 5. Conclusions

The chlorophyll content was increased accompanied with the exogenous N application, thus improving the photon assimilation. When compared to MN, LN decreased the chlorophyll content, Rubisco activity, and photon assimilation. These elements resulted in a significantly lower net photosynthetic rate in LN than MN. On the contrary, HN increased the chlorophyll content and photon assimilation. However, the net photosynthetic rate did not increase as expected. No significant difference was observed in Rubisco activity and NPQ between the HN and MN treatments for both cultivars. Excessive energy could not be dissipated by NPQ and this led to oxidative stress. The higher MDA content and H_2_O_2_ content in HN than in MN indicated the likelihood of photoinhibition, especially in Zhefu802. Therefore, we conclude that a high N supply does not allow for the ideal net photosynthetic rate and increases the likelihood of photoinhibition because of the unbalance of light absorption and utilization. Furthermore, NPQ may contribute to this imbalance. Our research provided a point of view exploring N use efficiency following light utilization balance.

## Figures and Tables

**Table 1:** Effect of nitrogen treatments on the content of pigments involved in the xanthophyll cycle. V, Violaxanthin; A, Antheraxanthin; Z, Zeaxanthin. LN, low nitrogen, 10 mg L^−1^; MN, moderate nitrogen, 40 mg L^−1^; HN, high nitrogen, 160 mg L^−1^. Different letters denote significant difference as determined by LSD test (*p* < 0.05).

Variety	Zhefu802			*Fgl*		
Treatment	LN	MN	HN	LN	MN	HN
V (μmol/mmol Chl)	47.05c	42.71d	41.42e	57.36a	53.55b	52.93b
A (μmol/mmol Chl)	30.40c	23.73d	16.64e	40.13a	35.32b	29.42c
Z (μmol/mmol Chl)	158.23a	141.39b	135.17c	142.39b	130.48d	126.57e
β-Carotene (μmol/mmol Chl)	438.21c	431.95cd	420.40d	645.83a	582.93b	596.31b
Z + 1/2A	173.44a	153.26c	143.49e	162.46b	148.14d	141.28e
